# Substrate stiffness and pressure alter retinal Müller glia response and extracellular matrix production

**DOI:** 10.1016/j.bbiosy.2025.100114

**Published:** 2025-07-07

**Authors:** Laura Prieto-López, Xandra Pereiro, Emilio J. González Ramírez, Noelia Ruzafa, Alicia Alonso, Kristian Franze, Elena Vecino

**Affiliations:** a Experimental Ophthalmo-Biology Group, Department of Cell Biology and Histology, University of Basque Country UPV/EHU, 48940 Leioa, Spain; b Begiker-Ophthalmology Research Group, BioCruces Health Research Institute, Cruces Hospital, 48903 Barakaldo, Spain; c Instituto Biofisika (UPV/EHU, CSIC), Department of Biochemistry and Molecular Biology, University of the Basque Country UPV/EHU, 48940 Leioa, Spain; d Department of Physiology, Development and Neuroscience, University of Cambridge, Cambridge, CB2 3DY, UK; e Institute of Medical Physics and Micro-Tissue Engineering, Friedrich-Alexander Universität Erlangen-Nürnberg, 91054 Erlangen, Germany; f Max-Planck-Zentrum für Physik und Medizin, 91054 Erlangen, Germany

**Keywords:** Müller glia, Extracellular matrix, Polyacrylamide gels, Stiffness, Pressure, TGF-β1

## Abstract

**Background:**

The retina is highly influenced by its mechanical environment, with Müller glia (MG) acting as key mechanosensors and extracellular matrix (ECM) producers. This study examined MG responses to substrate stiffness and high pressure (HP), and whether TGF-β1 modulation could mitigate these effects.

**Methods:**

Primary MG from adult rat retinas were cultured on glass (Young’s modulus E’=**∼**1 gigapascal (GPa)) and polyacrylamide gels (10 kPa and 100 kPa). MG were exposed to atmospheric and 70 mmHg (HP) conditions, with TGF-β1 pharmacologically blocked.

**Results:**

On glass and 100 kPa gels, MG survival, cell area, and ECM deposition (collagen I, IV, and fibronectin) increased, with cells adopting a fusiform shape and more dedifferentiated state. Under HP, survival decreased on stiffer substrates, though cell area and morphology remained unchanged. HP increased ECM deposition, which was reduced by TGF-β1 inhibition.

**Conclusions:**

Our findings suggest that MG response to mechanical stress alter their survival and cell area, and increases ECM secretion, highlighting TGF-β1 as a potential therapeutic target.

## Highlights

• Müller glia survival and extracellular matrix deposition increase on stiffer substrates.
• High pressure reduces Müller glia survival but increases matrix deposition.
• Inhibition of TGFβ1 reduces extracellular matrix deposition.

## Graphical abstract

**Figure:**
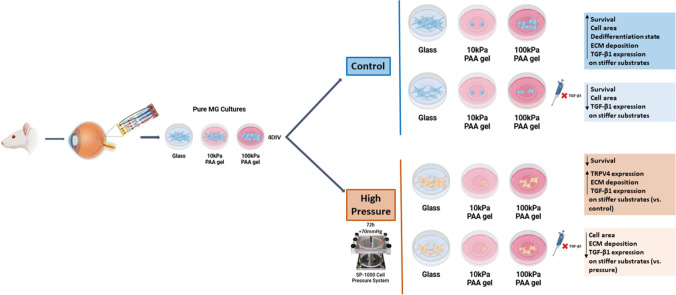

## Introduction

In recent years, the biophysical environment of tissues and particularly the stiffness of its extracellular matrix (ECM) has emerged as a key regulator of cell behavior. The retina is a highly mechanically heterogeneous tissue, constantly subjected to mechanical cues [1]. Thus, retinal ECM stiffness plays a key role in both physiological and pathological states [2]. During retinal development, the ECM stiffness mediates cell layer organization and cell differentiation and maturation [[3], [4], [5]]. However, in many retinopathies the ECM undergoes remodeling with abnormal deposition of ECM components [[6], [7], [8], [9]].

Müller glia (MG) are the main glial cell type in the retina, spanning its entire thickness and making contact with all retinal neuron cell types. Initially thought to provide only structural support, MG are now recognized as key players in retinal homeostasis. They participate in various processes, such as metabolism and waste removal [10], neuronal signaling [11], water and ion homeostasis [12], and the production of neurotrophic factors [13,14], among others. In addition to their biochemical roles, MG are essential in maintaining the mechanical homeostasis of the retina.

MG both provide tensile strength to keep tissue integrity thanks to their morphology [15,^16^] but also serve as the retina's primary mechanosensors [17]. They respond to mechanical changes by locally adapting their structure and function to support surrounding neurons [13,18]. For instance, our group has demonstrated that MG respond differently to high pressure (HP) conditions, a hallmark of glaucoma, depending on their location in the retina, with peripheral MG being more susceptible to HP [19]. Interestingly, chronic elevated intraocular pressure (IOP) in glaucoma leads to excessive ECM deposition, resulting in substrate stiffening [20]. The stiffened ECM exerts increasing tensile forces on MG, which sense these changes through different mechanosensitive channels such as the polymodal channel TRPV4 (transient receptor potential vanilloid 4), whose activation triggers MG gliosis and the release of pro-inflammatory cytokines [21], and the ion channel Piezo1, which optimizes the responsiveness of retinal glial populations [22].

MG are major producers of ECM components, such as fibronectin, collagens, and laminin, among many others, which are key in retinal architecture [23]. For example, fibronectin is required to maintain the composition of cell–matrix adhesion sites and acts as scaffolding for the collagen network [24]. Meanwhile, collagen I and collagen IV are vital for the integrity of the retinal ganglion cell layer (GCL) and the inner limiting membrane (ILM), respectively [25,26]. In the genetic PTP-Meg2 HET murine glaucoma model, MG mediated deposition contributed to tissue stiffening [27], further promoting MG reactivity. This process reflects a broader phenomenon observed during retinal degeneration, where MG become reactive, undergo gliosis, and contribute to ECM remodeling and mechanical stiffening of the retina. Among the key mediators of this response, the cytokine Transforming Growth Factor **(**TGF)-β1 plays a central role in orchestrating the transition to a reactive, fibrotic state**,** promoting ECM production in a feed-forward loop [28,29].

Understanding how MG respond to and influence mechanical cues, and the extent to which TGF-β1 contributes to this feedback loop, is crucial for identifying therapeutic strategies in diseases such as glaucoma and proliferative vitreoretinopathy.Primary MG culture is an essential model to study the physiology and pathophysiology of these cells under controlled conditions. Conventional cell cultures are performed on hard materials, such as plastic or glass, with a Young’s modulus (E’; a mechanical property that measures the stiffness of materials) of **∼**1 gigapascal (GPa), which is significantly higher than the retina's physiological stiffness. Although retinal stiffness values vary significantly depending on the method, species, and retinal region analysed, it is in the range of kPa. For example, Lu et al. [30] reported a bulk stiffness of 0.1 kPa in guinea pig retinal explants. Later on, the same group measured a range between 0.9–2.1 kPa depending on distance from the optic nerve head and regional localization [1]. Additional studies conducted by Qu et al. found retinal stiffnesses ranging from 1.3 to 26 kPa across layers of porcine explants [31] and a 3–16 kPa range in an in vivo rabbit model [32]. All of these experiments mimicked physiological conditions; no studies to our knowledge have directly measured stiffness in glaucomatous retinas. However, other ocular tissues implicated in glaucoma (e.g., trabecular meshwork, Schlemm’s canal) show a 1.5–20-fold increase in stiffness [[33], [34], [35]] and we hypothesize the retina follow a similar pattern.

Therefore, it is crucial to study MG behavior on substrates that better replicate the retinal environment. To address this issue and based on our experience in handling MG in culture [36], we first studied the behavior of MG grown on polyacrylamide (PAA) gels of different stiffnesses: a soft (physiological) and a hard (pathological) gel. PAA gels were chosen for their inert, biocompatible, non cytotoxic characteristics and ease of tuning their stiffness [37]. To mimic pathological conditions, we examined the effect of HP on MG cultures and investigated the potential modulation of MG response by inhibiting TGF-β1 expression. Thus, this study explores the impact of substrate stiffness on MG behavior under both control and HP conditions and evaluates TGF-β1 inhibition as a potential target for modulating MG mechanical responses.

## Results

### PAA gels Young’s moduli

Given MG role in retinal ECM maintenance and that the physical characteristics of the substrate are key in cell survival, area and morphology, we first performed MG cultures on PAA gels of different stiffnesses, and glass (i.e. conventional culture). We confirmed the gels nominal stiffness (a soft 10 kPa gel and a hard 100 kPa gel) by AFM, obtaining Young’s modulus (E’) values in agreement with nominal ones (Table 1).

**Table 1:** Measure of the PAA gel stiffness. Gel stiffness was measured by AFM. Results were given as the substrate’s Young’s modulus (E’).

Gel	Nominal E’ (kPa)	Measured E’ (mean kPa ± SEM)	Independent measures
Soft	10	10.6 ± 1.3	10
Hard	100	101.8 ± 6.4	10

### MG survival and cell area is enhanced on stiffer substrates

**Fig. 1:**
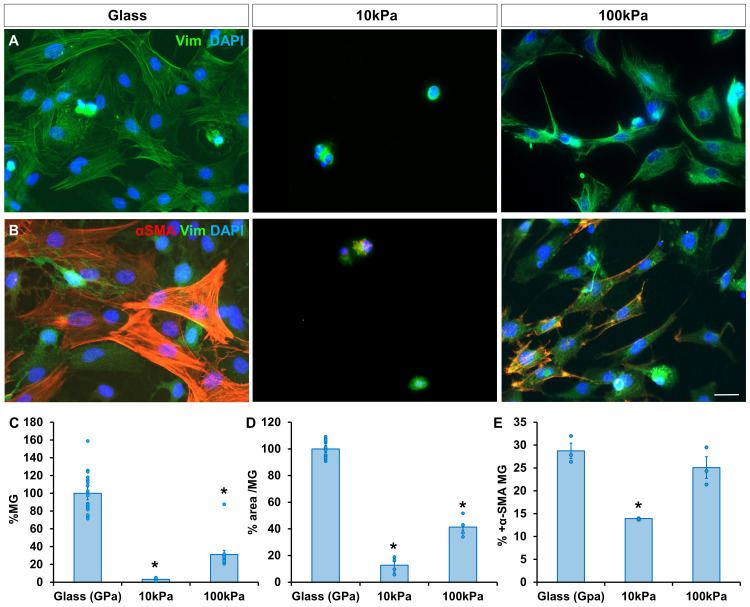
MG behavior on substrates of different stiffnesses. (A) Survival, cell area and morphology of MG cultured on PAA gels of 10 kPa and 100 kPa, and glass. The same number of MG were seeded on all substrates. (B) Immunolabelling of α-SMA in MG cultured on substrates of different stiffnesses. (C, D) Histograms of the percentage of MG and their cell area in the cultures on PAA gels relative to glass. (E) The percentage of MG positive to α-SMA in each substrate is shown. MG were labeled with antibodies against vimentin (green) and α-SMA (red). Nuclei were stained with DAPI (blue). Data is represented as mean ± SEM. *p-value < 0.05. Scale bar: 20 μm.

Considering that the physical characteristics of the substrate are key in cell survival, area and morphology, the number of MG cultured on substrates with different stiffness levels was analyzed. There were less cells on PAA gels compared to glass. Still, MG numbers were higher on the 100 kPa gel (31.08 ± 4.81 %) compared to the 10 kPa one (3.13 ± 0.57 %) (Fig. 1A). The proliferative profile of MG on the different substrates (see Supplementary Fig. 1) suggests that this behavior is caused by higher cell survival on stiffer gels and not their proliferative capacity.

Additionally, MG cell area was significantly higher when cultured on glass, with cells exhibiting a more fusiform morphology. In contrast, cells on soft PAA gels adopted a different phenotype compared to those on the stiffer substrate. MG cultured on 10 kPa gels appeared rounded with a substantially reduced cell area (12.72 ± 5.24 %), whereas cells on the 100 kPa gel displayed a more spread-out morphology, resembling cells grown on glass but with a still reduced cell area (41.39 ± 3.89 %) (Fig. 1A).

### MG are more dedifferentiated on stiffer substrates

To assess the dedifferentiation state of MG, the expression of α-SMA (smooth muscle actin), a marker of dedifferentiation, was evaluated. A similar percentage of MG expressed α-SMA when cultured on glass (28.75 ± 1.69 %) and 100 kPa gels (25.07 ± 2.37 %), whereas MG on 10 kPa gels showed lower α-SMA expression (13.91 ± 0.05 %), suggesting that MG cultured on stiffer substrates tend to be more dedifferentiated (Fig. 1B).

### HP reduces MG survival but has no effect on cell area and morphology

**Fig. 2:**
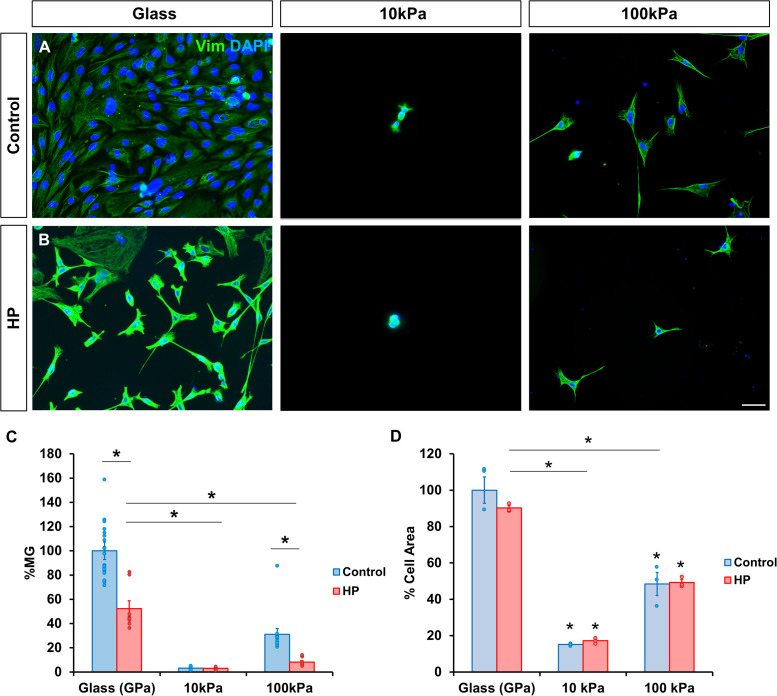
HP conditions reduce MG survival but has no effect on cell area and morphology. Survival and morphology of MG cultured on PAA gels of 10 kPa and 100 kPa, and glass (A) in atmospheric conditions and (B) after being subjected to HP for 72 h. The same number of MG were seeded in all substrates. (C) Histogram of the percentage of MG in the cultures on PAA gels relative to glass. MG were labeled with antibody against vimentin (green). Nuclei were stained with DAPI (blue). Data is represented as mean ± SEM. *p-value < 0.05. Scale bar: 50 μm.

Since elevated IOP has been linked to MG reactivity and increased retinal stiffness, the effect of HP on MG survival was analyzed across the different substrates. After 72 h under HP, MG survival on glass decreased to 52.32 ± 6.48 %. Likewise, the survival of MG cultured on 100 kPa gels dropped to 8.22 ± 1.18 %, compared to 31.08 ± 4.81 % under atmospheric conditions. Interestingly, cells cultured on 10 kPa gels were minimally affected by HP, with survival rates remaining similar (3.01 ± 0.45 % under HP vs. 3.13 ± 0.57 % in control conditions) (Fig. 2A and B). Notably, cell area and morphology were primarily influenced by substrate stiffness rather than HP.

### HP increases MG expression of mechanoreceptor TRPV4 on stiffer substrates

**Fig. 3:**
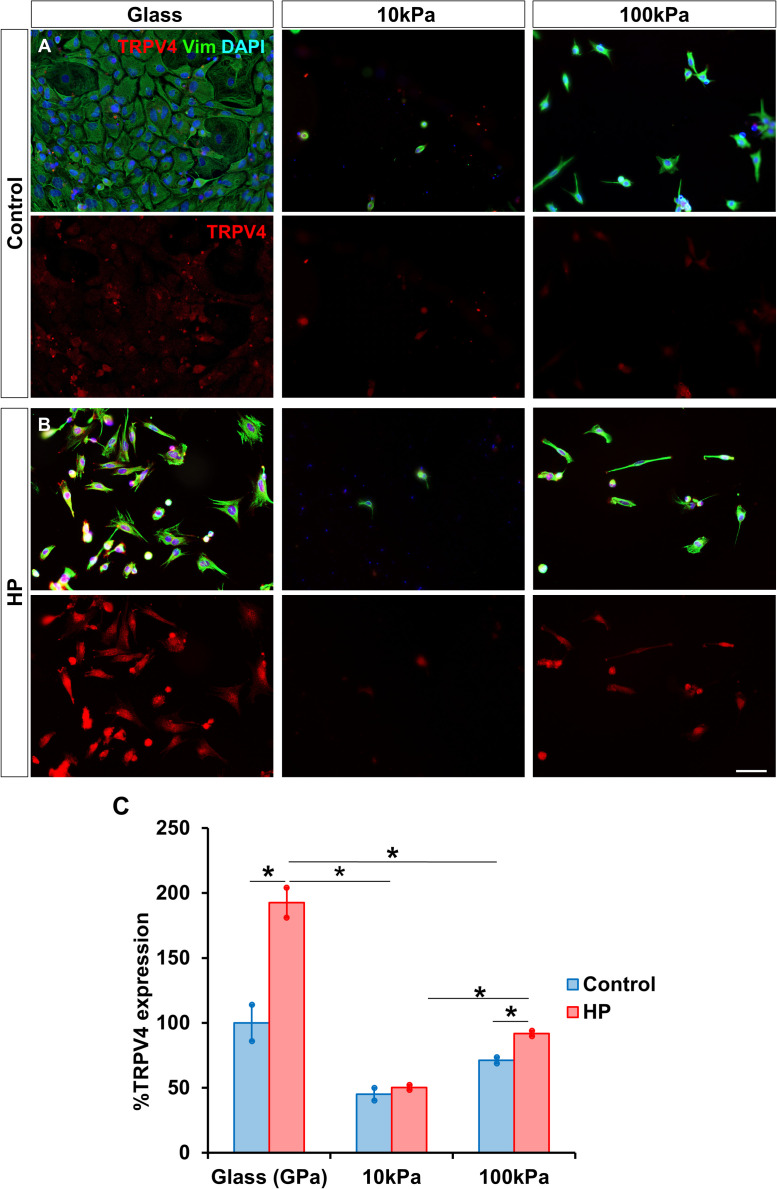
HP increases MG expression of mechanoreceptor TRPV4 on stiffer substrates. Expression of the mechanosensitive channel TRPV4 by MG cultured on PAA gels of 10 kPa and 100 kPa, and glass (A) in atmospheric conditions and (B) after being subjected to HP for 72 h. The same number of MG were seeded in all substrates. (C) The percentage of TRPV4 expressed by MG relative to glass under atmospheric pressure condition. MG were labeled with antibody against vimentin (green) and TRPV4 (red). Nuclei were stained with DAPI (blue). Data is represented as mean ± SEM. *p-value < 0.05. Scale bar: 50 μm.

Furthermore, the expression of the mechanosensitive channel TRPV4 was also evaluated (Fig. 3). Under atmospheric conditions, TRPV4 expression did not significantly differ between substrates (Fig. 3A). However, under HP, TRPV4 expression significantly increased with substrate stiffness, reaching 192.62 ± 1.58 % on glass (vs. 100.00 ± 13.99 % in control) and 91.86 ± 6.15 % on 100 kPa gels (vs. 71.16 ± 2.33 % in control), while no significant changes were observed in the 10 kPa gels (Fig. 3B).

### HP and stiffer substrates promote MG deposition of ECM components

To assess the impact of HP on MG-driven ECM production, the deposition of collagen I, collagen IV, and fibronectin was analyzed.

**Fig. 4:**
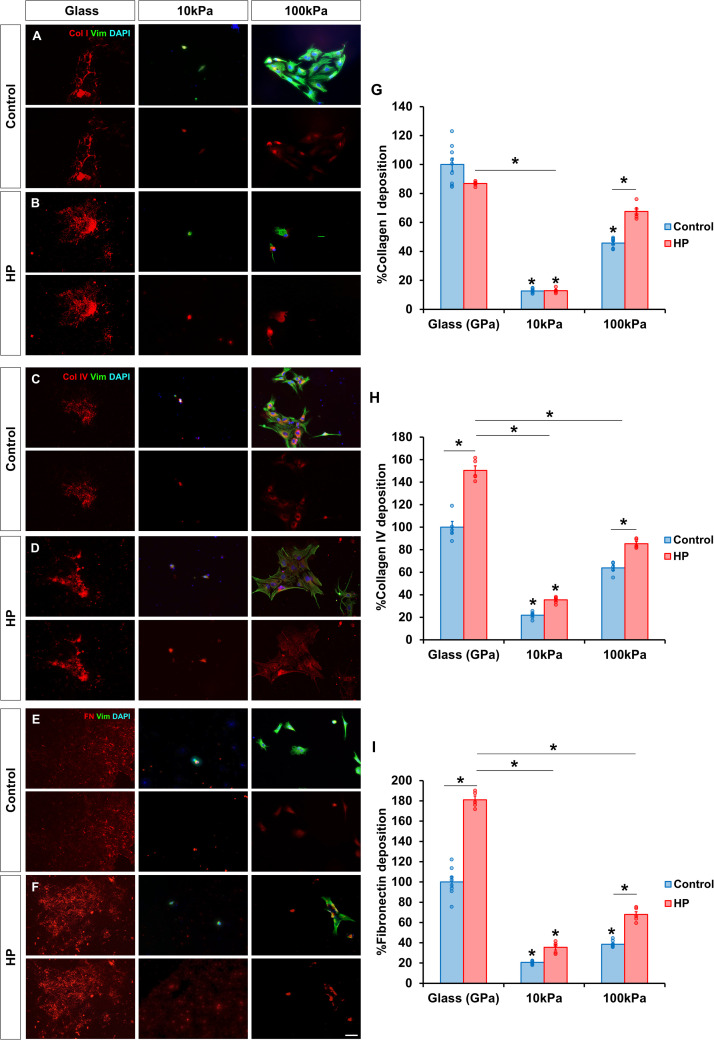
HP and stiffer substrates promote MG deposition of ECM components. Deposition of collagen I, collagen IV and fibronectin by MG under atmospheric conditions (A, C and E, respectively) and after being subjected to HP for 72 h (B, D and F, respectively). The same number of MG were seeded in all substrates. Prior to immunolabelling, MG from the glass control condition were removed to ensure that only deposited ECM was taken into account. Histogram of the percentage of (G) collagen I, (H) collagen IV and (I) fibronectin deposited by MG relative to glass under atmospheric pressure. MG were labeled with antibody against vimentin (green), collagen I (red, A,B), collagen IV (red, C, D) and fibronectin (red, E, F). Nuclei were stained with DAPI (blue). Data is represented as mean ± SEM. *p-value < 0.05. Scale bar: 50 μm.

For collagen I, stiffer substrates promoted greater deposition. MG grown on 10 kPa and 100 kPa gels deposited significantly less collagen I (12.70 ± 0.47 % and 45.79 ± 1.09 %, respectively) compared to those on glass (Fig. 4A). Under HP, collagen I deposition on glass showed a decreasing trend (86.90 ± 0.74 %), while in the 100 kPa gel it significantly increased to 67.60 ± 2.43 % (Fig. 4B). Deposition on the 10 kPa gel remained unchanged.

The deposition of collagen IV was also influenced by the substrate stiffness; cells on 10 kPa gels and 100 kPa gels deposited less collagen IV (21.90 ± 1.48 % and 63.89 ± 2.44 %, respectively) compared to those on glass (Fig. 4C). Under HP conditions, collagen IV deposition increased across all substrates, reaching 150.41 ± 4.09 % on glass, 35.47 ± 1.31 % on 10 kPa gels and 85.33 ± 1.84 % on 100 kPa gels (Fig. 4D).

Fibronectin deposition mirrored the patterns of collagen I and IV. MG on glass deposited the most fibronectin, with lower levels observed on 10 kPa (20.62 ± 0.64 %) and 100 kPa (38.58 ± 1.14 %) gels (Fig. 4E). Under HP, fibronectin deposition increased significantly on glass (185.01 ± 7.45 %), while deposition on 100 kPa gels was also enhanced (67.89 ± 2.73 %) (Fig. 4F).

### TGF-β1 inhibition reduces MG survival and cell area under atmospheric conditions, but only cell area under HP

Given the association of TGF-β1 with MG response to stiffness, its expression and the effect of its inhibition were analyzed.

**Fig. 5:**
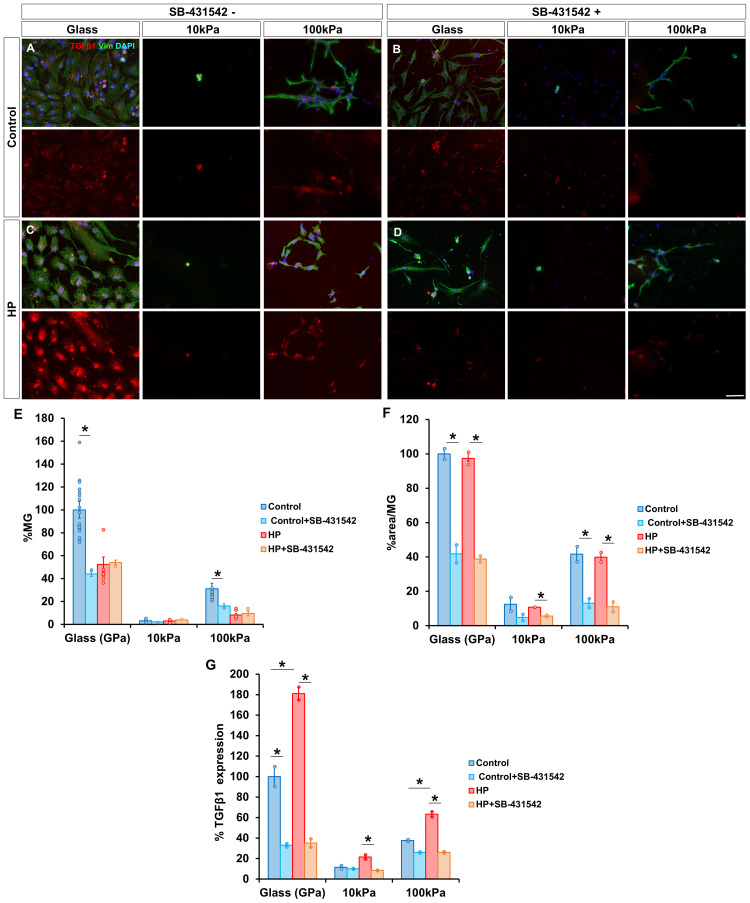
TGF-β1 inhibition reduces MG survival and cell area under atmospheric conditions, but only cell area under HP. Survival, morphology and expression of TGF-β1 of MG cultured on PAA gels of 10 kPa and 100 kPa, and glass (A) in atmospheric conditions, (B) after being subjected to HP for 72 h, and after treatment with 10μg/mL of the TGF-β1 inhibitor SB-431542 (C) under control and (D) HP conditions. The same number of MG were seeded in all substrates. (E, F) Histograms of the percentage of MG and their cell area in the cultures on PAA gels relative to glass. (G) The percentage of TGF-β1 expressed by MG relative to untreated glass under atmospheric pressure condition. MG were labeled with antibodies against vimentin (green) and TGF-β1 (red). Nuclei were stained with DAPI (blue). Data is represented as mean ± SEM. *p-value < 0.05. Scale bar: 50 μm.

Cells treated with the TGF-β1 inhibitor SB-431542 for 72 h exhibited a significant reduction in survival under atmospheric conditions. MG survival on glass decreased to 44.02 ± 2.12 % (vs. 100 ± 7.22 % untreated), and on 100 kPa gels, survival dropped from 31.08 ± 0.15 % to 16.14 ± 1.81 % (Fig. 5A and B). Under HP, TGF-β1 inhibition did not significantly affect MG survival (Fig. 5C and D).

Cell area was also significantly reduced by TGF-β1 inhibition across substrates under both conditions. For MG cultured on glass at atmospheric pressure, cell area reduced to 41.81 ± 5.23 % (vs. 100 ± 3.17 % for non SB-431542 treated cells), while on 100 kPa gels reduced to 13.10 ± 2.65 % (vs. 41.62 ± 4.49 %) and on 10 kPa gels to 4.82 ± 2.65 % (vs. 12.44 ± 4.02 %) (Fig. 5A and B). Similarly, when subjected to HP cell area for cells cultured on glass decreased to 38.83 ± 1.72 % (vs. 97.38 ± 3.60 %), for cells grown on 100 kPa gels to 11.02 ± 2.86 (vs. 39.88 ± 2.83 %) and for those grown on 10 kPa gels to 3.01 ± 0.49 % (vs. 10.75 ± 0.02 %) (Fig. 5C and D). Interestingly, MG treated with SB-431542 displayed similar cell area within the same substrate, regardless of pressure conditions.

TGF-β1 expression increased with the stiffness of the substrate; for MG in control conditions, TGF-β1 expression reduced to 11.42 ± 1.71 % and 37.69 ± 0.87 % on 10 kPa and 100 kPa gels, respectively, compared to glass (Fig. 5A). HP increased TGF-β1 expression; for cells cultured on glass reached 181.09 ± 6.31 %, while for MG on 10 kPa and 100 kPa gels raised to 21.52 ± 2.13 % and 63.23 ± 2.60 %, respectively (Fig. 5C). Treatment of MG with SB-431542 effectively reduced the cytokine expression. At atmospheric pressure, TGF-β1 expression reduced to 33.06 ± 1.69 % and 26.06 ± 0.58 % for MG grown on glass and 100 kPa gels, whereas expression on 10 kPa gels did not significantly changed (Fig. 5A and B). Under HP, TGF-β1 expression reduced to 35.15 ± 4.13 %, 8.44 ± 0.37 % and 26.03 ± 0.93 % on glass, 10 kPa gels and 100 kPa gels, respectively (Fig. 5C and D). Cells treated with TGF-β1 inhibitor showed similar expression levels within the same substrate under both pressure conditions.

### TGF-β1 inhibition reduces MG deposition of ECM components under HP on stiffer substrates

As we have established that MG differently deposited ECM, we analyzed the effect of inhibiting TGF-β1 on MG deposition.

**Fig. 6:**
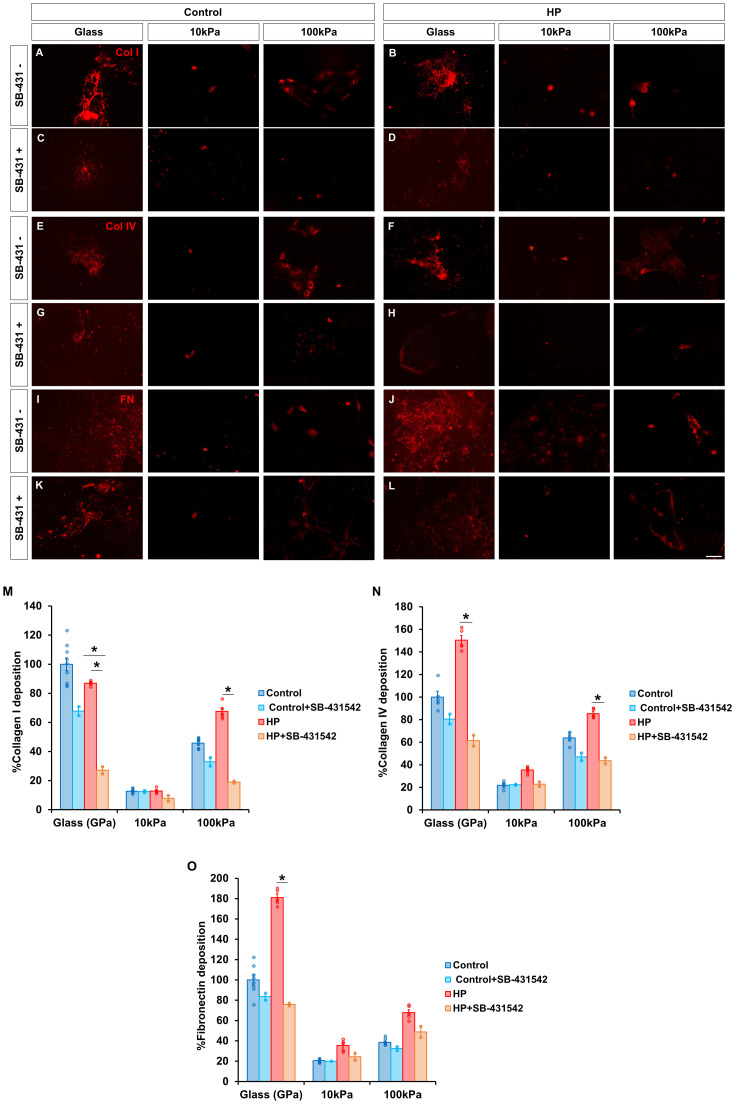
TGF-β1 inhibition reduces MG deposition of ECM components under HP on stiffer substrates. Deposition of (A-D) collagen I, (E-H) collagen IV and (I-L) fibronectin by MG at (A,E,I) atmospheric pressure and (B,F,J) after being subjected to HP for 72 h, and after treatment with 10μg/mL of the TGF-β1 inhibitor SB-431542 (C,G,K) under control and (D,H,L) HP conditions. The same cell number was seeded in all substrates. Prior to immunolabelling, MG from the glass control condition were removed to ensure that only deposited ECM was taken into account. Histogram of the percentage of (M) collagen I, (N) collagen IV and (O) fibronectin deposited by MG relative to untreated glass under atmospheric pressure. Only deposition is shown for simplifying purposes. MG were labeled with antibody against collagen I (red, A-D), collagen IV (red, E-H) and fibronectin (red, I-L). Data is represented as mean ± SEM. *p-value < 0.05. Scale bar: 50 μm.

At atmospheric pressure, inhibiting TGF-β1 expression did not significantly change the deposition of collagen I, collagen IV and fibronectin (Fig. 6A, C, E, G, I and K). When MG were subjected to HP, deposition of collagen I on glass (27.13 ± 2.46 % vs. 86.90 ± 0.74 %) and on 100 kPa gels (18.96 ± 0.75 % vs. 67.60 ± 2.43 %) was significantly reduced by treating cells with SB-431542 (Fig. 6B and D). Likewise, when TGF-β1 expression was inhibited there was a significant reduction in collagen I deposition between MG cultured on glass at atmospheric pressure (67.77 ± 3.05 %) and at HP (27.13 ± 2.46 %) (Fig. 6C and D).

Deposition of collagen IV also significantly decreased in HP conditions; when TGF-β1 expression was inhibited, collagen IV deposition on glass reduced to 61.41 ± 4.83 % (vs.150.41 ± 4.09 %) while on 100 kPa decreased to 43.63 ± 2.76 % (vs. 85.33 ± 1.84 %) (Fig. 6F and H). Interestingly, SB-431542 treated MG deposition of collagen IV was similar within the substrate, regardless of pressure conditions.

Fibronectin deposition was also significantly decreased on glass under HP conditions (75.88 ± 1.21 % vs. 181.01 ± 3.75 % untreated) (Fig. 6J and L). Similarly to collagen IV deposition, secretion of fibronectin was similar within the substrate, regardless of pressure conditions.

## Discussion

The retina is a tissue highly exposed to mechanical inputs [1], where ECM and tissue stiffness play key roles in its homeostasis. As MG are the only cells spanning the entire thickness of the retina, they are exposed to significant forces [15]. Accordingly, MG have been identified as a key mechanosensitive cell type in the retina [17] and major ECM producer [23]. Therefore, gaining a better understanding of how MG respond to changes in substrate stiffness would shed light in retinal physiology and pathophysiology. However, conventional cultures are typically performed on hard materials, with stiffness levels several orders of magnitude higher than those of the native retina [30]. This highlights the importance of using in vitro models that better replicate the mechanical properties of the in vivo environment to improve our understanding of retinal physiology. Here, we analyzed the susceptibility of MG to substrate stiffness in vitro and its relation to HP, a pathological mechanical stimulus primarily associated with glaucoma. To achieve this, we utilized PAA gels [38], which provide tunable stiffness and allowed us to study MG within a range of stiffness values found in the native retina [1,[30], [31], [32]].

Our data indicate that primary adult MG exhibit distinct behaviors depending on substrate stiffness in vitro. Surprisingly, MG exhibited reduced survival and cell area on the more physiologically relevant 10 kPa gels, promoting a rounded morphology. Similar findings have been reported in the spontaneously immortalized human MG cell line MIO-M1 [7], as well as other cell types such as fibroblasts [39]. This behavior can likely be attributed to the tractional forces MG exert on the substrate. On the compliant 10 kPa gels MG displayed low cytoskeletal tension, failing to generate stress fibers and exert strong tractional forces [40]. Indeed, other glial cell types such as microglia, adapt their spread area, morphology, and actin cytoskeleton to the stiffness of their environment, exerting higher traction forces on stiffer substrates [38,41]. Since MG require specific mechanical tension to stretch and maintain retinal integrity [1,30], stiffer substrates likely provide the necessary mechanical input for MG cells to spread and survive. Furthermore, our analysis of MG proliferation (see Supplementary Fig. 1) suggests that MG viability on softer substrates is reduced. A higher percentage of proliferating MG were found on the 10 kPa gels in comparison with stiffer substrates, suggesting that only the more active cells in these gels, the ones attempting to proliferate, are the small subset of cells that survive. However, the increased survival of MG on stiffer substrates in vitro should not be taken as direct evidence that MG are more prone to dying in their native, softer environment. Rather, it highlights that in an in vitro setting, where additional stimuli are absent, softer substrates may not fully support MG survival or spreading. The native retinal microenvironment, with its complex ECM organization and interactions with neighboring cells, likely provides essential mechanical cues and growth factors that support MG survival and function even in softer, more compliant conditions.

Additionally, we observed that MG cultured on stiffer substrates (glass or 100 kPa gels) exhibited higher expression of α-SMA, consistent with previous studies in the MIO-M1 cell line [42], microglia [38] and astrocytes [37]. Particularly on glass, α-SMA, which integrates into stress fibers, was highly organized, potentially contributing to the observed cell morphology on stiff substrates. A stiff environment promotes α-SMA upregulation and organization in MG, leading to their elongation and adoption of a more dedifferentiated, myofibroblast-like state [43,44].

To simulate a pathological environment, we exposed MG to HP conditions, as elevated IOP is a key factor in glaucoma and has been shown to influence ECM stiffness [20,27]. Our results indicate that HP exposure reduced MG survival on stiff substrates (both glass and 100 kPa gels). To further investigate the effects of HP, we evaluated the expression of the mechanoreceptor TRPV4, a non-selective polymodal cation channel linked to MG reactivity and retinal cell apoptosis [45]. While no significant differences were observed under atmospheric conditions, TRPV4 expression increased under HP on stiff substrates, suggesting that MG sense pressure changes, but not substrate stiffness, via TRPV4. These findings align with studies in HEK-293T cells, which demonstrated that substrate stiffness had minimal effects on TRPV4 activation, requiring larger stimuli for activation [46]. Indeed, for MG on 10 kPa gels, where we did not observe significant differences in cell survival when subjected to HP, there was no significant variation in TRPV4 expression either, which may imply that MG in more compliant substrates stay in a quiescent state, less sensitive to mechanical stimuli. Interestingly, MG morphology and cell area were influenced by substrate stiffness but not by HP, highlighting the role of ECM in MG organization.

As primary ECM producers, we analyzed MG deposition of key retinal ECM components collagen I, collagen IV and fibronectin. Our results showed increased deposition of all three components on stiffer substrates, with further increases under HP, while no significant changes were observed on 10 kPa gels. This suggests that softer substrates reduce MG responsiveness to pressure. Under HP on glass, collagen I deposition tended to decrease compared to atmospheric conditions. Conflicting reports exist regarding collagen I deposition in the central nervous system under stress; some studies link high IOP to increased collagen I deposition in the GCL and optic nerve head [20], while others report reduced deposition of collagen I during glial scar formation [47]. In glial scars of the CNS, which are soft, collagen I is largely absent, while collagen IV participates in early stages of the scarring process. This could be attributed to collagen I forming stiff, compact fibers, whereas collagen IV (and fibronectin) create stretchable meshworks that stiffen the tissue to a lesser degree, allowing for ECM reorganization [48,49]. Our findings suggest that on extremely stiff substrates such as glass, MG may prioritize ECM remodeling over stiffening, leading to reduced collagen I deposition. On 100 kPa gels —closer to pathological in vivo conditions—they increase the production of both collagen types. This response may reflect an adaptive strategy to protect retinal ganglion cells (RGCs), which are particularly vulnerable in glaucoma [50]. Given that RGCs thrive in softer, more compliant substrates [30], MG may reduce collagen I deposition to preserve a neuroprotective environment.

MG reactiveness is tightly linked to biomechanical changes in the tissue, notably increased retinal stiffness, which further perpetuates glial activation. Substrate stiffness has been shown to induce ECM deposition through transforming growth factor β (TGF-β), specifically its profibrotic isoform TGF-β1, the most prevalent in the mammal retina [51]. TGF-β1 signaling promotes the transcription of ECM proteins, including collagens and fibronectin, as well as regulators of cytoskeletal tension, thereby reinforcing a positive feedback loop between MG activation and tissue stiffening [52]. Increased IOP is also known to activate TGF-β1 [53] and generate traction forces that stiffen the substrate in vivo [27], which our data support. Therefore, to further investigate the role of TGF-β1, we pharmacologically inhibited it using SB-431542, a selective TGF-β inhibitor [54].

Under atmospheric conditions, inhibition of TGF-β1 significantly reduced MG survival on stiff substrates, whereas no effect was observed under HP conditions. Conflicting reports exist regarding TGF-β1 effect on cell proliferation in glial cells; some studies suggest a proliferative effect on microglia [55], while others suggest that microglia and astrocytes age- and environment-related desensitization to TGF-β1 promote their proliferation [56,57]. Therefore, it seems that the effect of TGF-β1 on cell survival is cell- and context-dependent, where it supports MG survival on stiff substrates not in the presence of other mechanical stimuli (i.e. pressure).

TGF-β1 has been shown to promote cell area spreading in different glial [58] and non-glial [59,60] cell types, and indeed TGF-β1 inhibition significantly reduced MG cell area across substrates, regardless of pressure conditions, further confirming that cell spreading is substrate-driven and mediated by TGF-β1.

Treating MG with the inhibitor SB-431,542 effectively reduced TGF-β1 expression in all conditions, showing similar expression levels within the same substrate. SB-431542 treatment did not affect deposition of ECM components (collagen I, collagen IV and fibronectin) at atmospheric pressure; other pro-fibrotic cytokines, such as the isoform TGF-β2, although less prevalent in the retina and with lower activity [51,61] also participate in ECM deposition, and may be compensating for the reduced TGF-β1 levels. However, deposition significantly decreased under HP conditions, which might imply that the alternative pathways were insufficient to maintain deposition. Interestingly, MG treated with the inhibitor deposited significantly less collagen I on glass under HP. The differential collagen type deposition could be due to alternative factors acting downstream of TGF-β1. It has been reported that epidermal growth factor (EGF) promotes collagen IV but not collagen I production, while tumor necrosis factor α (TNF-α) specifically suppresses collagen I deposition [62], but the exact characterization and role of these other factors in response to mechanical stimuli is yet to be determined.

## Conclusions

This study demonstrates that MG are sensitive to changes in substrate stiffness, based on their behavior and the deposition of ECM components. Stiffer substrates promote cytoskeletal tension and stress fiber formation, supporting cell spreading and survival, and ECM deposition. However, this enhanced tension also marks a shift toward a reactive, dedifferentiated state. Furthermore, MG grown on stiffer substrates appear to be "primed" for mechanosensitive signaling, in accordance to TRPV4 upregulation under high-pressure conditions, leading to enhanced ECM deposition. These data suggest that stiffness orchestrates a coordinated cellular response through cytoskeletal regulation, linking morphology, viability, phenotype, and mechanotransduction. MG mechanoresaponse is mediated by the activation of the pro-fibrotic cytokine TGF-β1, but a better understanding of alternative factors involved would be necessary to better modulate MG response to mechanical stimuli. We conclude that MG are important sensors of mechanical changes in the retina, that they respond to them mainly through the activation of TGF-β1, which arises as a good potential target to alleviate the pro-fibrotic behavior of MG in adverse mechanical environments.

## Methods

### Animals

Adult 2-month-old Sprague Dawley rat (*n* = 50) eyes were obtained from animals reared at the University’s animal house (University of the Basque Country, UPV/EHU). Animals were kept in standard housing conditions on a 12 h light-dark cycle, with ad libitum access to food and water. Rats were humanely sacrificed by exposure to CO_2_. This study was carried out in strict accordance with the recommendations in the Guide for the Care and Use of Laboratory Animals. The experimental protocol met European (2010/63/UE) and Spanish (RD53/2013) standards for the protection of experimental animals, and it was approved by the Ethical Committee for Animal Welfare of the University of Basque country.

### Isolation and culture of adult Müller glia

Rat eyes were dissected immediately after enucleation. The retina was isolated in fresh CO_2_-independent Dulbecco’s modified Eagle’s medium (DMEM/CO_2_; Gibco-Life Technologies, Waltham, MA, United States) by circumferential section of the cornea and removal of the anterior chamber.

MG cultures were obtained as described by Pereiro et al. [36]; this protocol yields pure MG cultures as assessed by immunocytochemistry and flow cytometry. Briefly, the retinas were incubated at 37 °C for 30 min in a Sterile Earle’s Balanced Salt Solution (EBSS) containing 20 U/mL Papain (Worthington, Lakewood, NJ, United States) and 2000 U/mL DNase (Worthington, Lakewood, NJ, United States). To stop the enzyme digestion, DMEM containing 10 % FBS (fetal bovine serum) was added for 3 min at room temperature, and the tissue was then dissociated mechanically by careful homogenizing with pipettes of different tip sizes, recovering the cells by centrifugation at 1200 rpm for 5 min. The pelleted cells were re-suspended in DMEM + 10 % FBS medium and seeded on either PAA gels or glass-bottomed culture dishes. The amount of the cells seeded was 2.5 × 10^6^ cells per dish. Both PAA gels and glass-bottomed dishes were coated with 100 μg/mL poly-l-lysine (PLL; Sigma-Aldrich, St. Louis, MO, United States) in distilled water overnight followed by 10 μg/mL laminin (Sigma-Aldrich, St. Louis, MO, United States) in Phosphate Buffer Saline (PBS; Gibco-Life Technologies, Waltham, MA, United States) for at least 2 h prior to cell seeding. The cultures were maintained in a humidified incubator at 37 °C in an atmosphere of 5 % CO_2_, 95 % O_2_. The medium was totally replaced with fresh medium on day 1 of culture. Subsequently, half of the medium was changed every 2 days until the cultures reached confluence after 7 days in vitro (DIV). The procedure was performed at least in triplicate for each independent experiment.

### PAA gel preparation

To test the response of MG to substrate stiffness, PAA gels of different stiffnesses were prepared on detachable glass-bottomed 35 mm Petri dishes (Ibidi, Gräfelfing, Germany), as previously described by Moshayedi et al. [37]. Briefly, the dishes were washed in 70 % ethanol and distilled water, and then air-dried. Afterwards, they were pre-treated with 0.1 M sodium hydroxide (NaOH), dried, and incubated for 3 min with 300 μL of (3-aminopropyl) trimethoxysilane (APMS; Sigma-Aldrich, St. Louis, MO, United States). APMS was washed off in distilled water and Petri dishes were then incubated with 500 μL of 0.5 % glutaraldehyde (Merck-Millipore, Darmstadt, Germany) for 30 min, rinsed with distilled water and air-dried. Likewise, 20 mm top coverslips were cleaned with 70 % ethanol and distilled water and functionalized with the hydrophobic agent polydimethylsiloxane (RainX; ITW Global Brands, Glenview, IL, United States) and left to air-dry.

For the preparation of the gels, the protocol described by Bollmann et al. [38] was used. 500 μL of 40 % acrylamide (Sigma-Aldrich, St. Louis, MO, United States) were mixed with 65 μL of hydroxyethyl-acrylamide (Sigma-Aldrich, St. Louis, MO, United States). Next, 250 μL of 2 % bis-acrylamide (Fisher Scientific, Hampton, NH, United States) were added to 500 μL of the prepared mix to obtain the premix gel. Gel solutions were prepared by mixing premix gel and PBS and subjected to vacuum conditions to de-gass for 7–10 min. For correct polymerization, 1.5 μL of *N,N,N,N’*-Tetramethyl ethylenediamine (TEMED; Life Technologies, Waltham, MA, United States) and 5 μL of 0.01 % ammonium persulfate (APS; Sigma-Aldrich, St. Louis, MO, United States) were added to the mix. A volume of 17 μL of the final mixture was then pipetted on the bottom coverslip and 20 mm top coverslips were placed on top to ensure the formation of an even gel layer. The gels were incubated in Dulbecco’s Phosphate Buffer Saline (DPBS; Gibco-Life Technologies, Waltham, MA, United States) for at least 10–15 minutes before the top coverslips were removed. Afterwards, PAA gels were rinsed twice in DPBS and then activated with ultraviolet light for 30 min. PAA gels were coated as previously described.In preliminary work, we tested substrates of 1, 10, 45, and 100 kPa (data not shown). No significant behavioural differences were seen between 1 and 10 kPa, and 10 kPa substrates proved more stable and easier to handle. Similarly, results on 45 kPa gels were inconclusive, while 100 kPa allowed yielded consistent responses of MG on stiff conditions. All subsequent work was conducted exclusively on the 10 kPa and 100 kPa gels, as reflected in the manuscript.

### Atomic force microscopy

To confirm that the PAA gels prepared have different stiffness for each condition, atomic force microscopy (AFM) was used to measure the Young’s modulus (E’) of PAA gels, on a Nanowizard CellHesion 200 AFM (JPK Instruments, Berlin, Germany) placed on an inverted microscope (Axio Observer A1, Zeiss) fitted with a motorized stage. A 5.0 μm borosilicate glass probe glued onto a round-tipped silicon nitride cantilever (Novascan Technologies, Boone, IA, United States) with a spring constant of 0.060 N/m was used. The force setpoint applied was of 10nN at a 5μm/s speed. 10 force–distance curves at least 100 different locations per gel were measured for each independent experiment (Fig. S2). Post-processing and analysis were done using JPK SPM data processing software, using the Hertz model to fit the approaching curve and extract the values of the substrate’s Young’s modulus. This model assumes purely elastic behavior and analyzes only the approach curve, disregarding any hysteresis between approach and retract curves that may indicate viscoelastic effects, allowing for effective comparison of relative gel stiffness across conditions.

### Cultures exposed to high pressure

To evaluate the response of MG to high pressure (HP), after 4DIV cultures were placed in a P-1000 cell pressure system (Strex Inc.; San Diego, USA). This set-up allows a constant pressure to be maintained with an air mixture of 95 % air, 5 % CO2 and 21 %O2. The pressure chamber was placed in a conventional incubator at 37 °C to maintain cells in a humidified environment and cultures were subjected to 70 mmHg above atmospheric pressure for 72 h. The pressure elevation was selected based on previous studies [63]. Although elevated IOP in glaucoma patients typically range between 20 and 35 mmHg, it requires extensive time to mimic this effect in vitro, compromising experimental outcome; a 70 mmHg pressure was reported to produce the most reliable and measurable effect on retinal cells in a relatively short time. Control cultures were kept at atmospheric pressure in a standard cell incubator until the end of the experiment at 7DIV, and at least three independent experiments were performed.

### TGF-β1 inhibition

To determine the effect of inhibiting TGF-β1 on MG activation, cells were treated with 10μg/mL of TGF-β1 inhibitor SB-431542 [64] (CaymanChemical, Ann Arbor, MI, United States) before subjecting them to HP for 72 h. Control cultures were also treated with 10μg/mL of SB-431542 and kept at atmospheric pressure in a standard cell incubator. At least three independent experiments were performed.

### Immunocytochemistry

After 7DIV, the cells were washed in PBS, fixed in methanol for 10 min at −20 °C and non-specific antigen binding was blocked for 30 min at room temperature with blocking buffer (0.25 % Triton X-100 and 3 % BSA-bovine serum albumin-in PBS). The primary antibodies used are shown in Table 2. The antibodies were diluted in blocking buffer and incubated with the cultures overnight at 4 °C with agitation. The cells were washed three times with PBS and the cultures were exposed for 1 h at room temperature to the corresponding secondary antibodies at a dilution of 1:1000 in blocking buffer: Alexa Fluor 488 and Alexa 555 conjugated goat anti-mouse and goat anti-rabbit antibodies (Invitrogen, Eugene, OR, United States) and counterstained with DAPI (4′,6-diamidino-2-phenylindole; Invitrogen, Eugene, OR, United States) at a dilution of 1:10,000. After three washes, the dishes were detached and resulting “coverslips” were mounted with PBS-glycerol (1:1).

**Table 2:** List of primary antibodies.

Antigen	Target	Host	Dilution	Supplier
α-SMA	Dedifferentiation/fibrosis	Mouse	1:1000	Sigma
Collagen I (col1a2)	ECM	Rabbit	1:500	Sigma
Collagen IV (col4a1)	ECM	Rabbit	1:500	Sigma
Fibronectin	ECM	Rabbit	1:500	Sigma
Ki67	Proliferation	Rabbit	1:500	Abcam
PCNA	Proliferation	Rabbit	1:500	Abcam
TGF-β1	Fibrosis	Rabbit	1:500	Abcam
TRPV4	Mechanosensor	Rabbit	1:500	LS-Bio
Vimentin	Müller glia	Mouse	1:1000	Dako
Vimentin	Müller glia	Rabbit	1:1000	Abcam

### BrdU integration assay

To corroborate the proliferation profile of MG among the different substrates, we performed a 5′‑bromo-2′-deoxyuridine (BrdU) integration assay. At 7DIV culture medium was replaced with 10µ M BrdU (Life Technologies, Waltham, MA, United States) in fresh medium. MG were incubated with the solution for 2 h. Cells were then washed with PBS, fixed in methanol for 10 min at −20 °C and incubated with blocking buffer for 30 min at room temperature. For BrdU staining, cells were pretreated with 1 N HCl for 10 min on ice followed by another 10-minute incubation with 2 N HCl at room temperature. MG were then washed with phosphate buffer (PB) and incubated in blocking buffer. From this point, cells were stained for vimentin as seen above; on day 2 of the protocol MG were incubated with mouse anti-BrdU-fluorescein antibody (1:200, Roche, Basel, Switzerland) for 1 h at room temperature, along with the corresponding secondary antibody for vimentin and counterstained with DAPI. After washing, the dishes were detached and mounted with PBS-glycerol (1:1).

### MG quantification

MG were analyzed on an epifluorescence microscope (Zeiss, Jena, Germany) coupled to a digital camera (Zeiss Axiocam MRM, Zeiss, Jena, Germany). Imaging acquisition was controlled by ZENpro software (Zeiss, Jena, Germany). To assess cell survival in each condition, MG were manually counted using ImageJ software (NIH, Bethesda, MD, United States). To analyze cell morphology, the cell contours were manually drawn using the Freehand Selections tool in ImageJ and subsequently cell area was automatically detected. The intensity of fluorescence of TRPV4 and TGF-β1 expressed by MG was measured using ImageJ as the integrated density of the fluorescence expressed by the cells [65]. Similarly, the intensity of fluorescence of the deposited ECM was also measured using ImageJ. In ECM deposition experiments, the integrated density was obtained after the fluorescence expressed by cells themselves was subtracted. Since on substrate stiffness studies Müller glia achieved confluence in the glass control condition, for deposition experiments cells were first removed by 5 min incubation with accutase (Sigma-Aldrich, St. Louis, MO, United States) prior to fixing in order to confidently analyze deposition and not intracellular expression. As MG did not reach confluence on PAA gels, ECM deposition could be confidently study without cell removal. All integrated density values were then normalized to cell survival.

### Statistical analysis

Experimental procedures were replicated at least three times for each experiment; at least 30 photos were taken for each condition. Results were normalized to control conditions: glass, atmospheric pressure, no TGF-β1 inhibition. The mean and standard error of mean (SEM) are presented for each condition. Statistical analyses were carried out using the SPSS Statistical software (IBM, Armonk, NY, United Sates). Data from the different experimental conditions were compared using a Kruskal–Wallis non-parametric test, followed by a post-hoc Dunn test. The minimum value of significance was defined as *p* < 0.05.

## CRediT authorship contribution statement

**Laura Prieto-López:** Writing – review & editing, Writing – original draft, Visualization, Methodology, Investigation, Formal analysis, Data curation. **Xandra Pereiro:** Writing – review & editing, Writing – original draft, Visualization, Validation, Supervision, Investigation, Formal analysis, Data curation, Conceptualization. **Emilio J. González Ramírez:** Writing – review & editing, Methodology. **Noelia Ruzafa:** Writing – review & editing, Visualization. **Alicia Alonso:** Writing – review & editing. **Kristian Franze:** Writing – review & editing, Methodology, Funding acquisition. **Elena Vecino:** Writing – review & editing, Writing – original draft, Visualization, Validation, Supervision, Project administration, Funding acquisition, Formal analysis, Data curation, Conceptualization.

## Declaration of competing interest

The authors declare that they have no known competing financial interests or personal relationships that could have appeared to influence the work reported in this paper.

## Data Availability

Data will be made available on request.

## Appendix Supplementary materials

Image, application 1

Image, application 2

## References

[1] Franze K., Francke M., Günter K., Christ A.F., Körber N., Reichenbach A. (2011). Spatial mapping of the mechanical properties of the living retina using scanning force microscopy. Soft Matter.

[2] Vecino E., Kwok J.C.F. (2016).

[3] Reinhard J., Joachim S.C., Faissner A. (2015). Extracellular matrix remodeling during retinal development. Exp Eye Res.

[4] Barriga E.H., Franze K., Charras G., Mayor R. (2018). Tissue stiffening coordinates morphogenesis by triggering collective cell migration in vivo. Nature.

[5] Segel M., Neumann B., Hill M.F.E., Weber I.P., Viscomi C., Zhao C. (2019). Niche stiffness underlies the ageing of central nervous system progenitor cells. Nature.

[6] Bringmann A., Wiedemann P. (2009). Involvement of Müller glial cells in epiretinal membrane formation. Graefes Arch Clin Exp Ophthalmol.

[7] Davis J.T., Wen Q., Janmey P.A., Otteson D.C., Foster W.J. (2012). Müller cell expression of genes implicated in proliferative vitreoretinopathy is influenced by substrate elastic modulus. Invest Ophthalmol Vis Sci.

[8] Mavlyutov T.A., Myrah J.J., Chauhan A.K., Liu Y., McDowell C.M. (2022). Fibronectin extra domain A (FN-EDA) causes glaucomatous trabecular meshwork, retina, and optic nerve damage in mice. Cell Biosci.

[9] Zhang W., Kong Y. (2020). YAP is essential for TGF-β-induced retinal fibrosis in diabetic rats via promoting the fibrogenic activity of Müller cells. J Cell Mol Med.

[10] Tsacopoulos M., Magistretti P.J. (1996). Metabolic coupling between glia and neurons. J Neurosci.

[11] Bringmann A., Pannicke T., Biedermann B., Francke M., Iandiev I., Grosche J. (2009). Role of retinal glial cells in neurotransmitter uptake and metabolism. Neurochem Int.

[12] Nagelhus E.A., Ottersen O.P. (2013). Physiological roles of aquaporin-4 in brain. Physiol Rev.

[13] García M., Forster V., Hicks D., Vecino E. (2002). Effects of Müller Glia on cell survival and neuritogenesis in adult porcine retina In vitro. Invest Ophthalmol Vis Sci.

[14] Ruzafa N., Pereiro X., Lepper M.F., Hauck S.M., Vecino E. (2018). A proteomics approach to identify candidate proteins secreted by Müller Glia that protect ganglion cells in the retina. Proteomics..

[15] MacDonald R.B., Randlett O., Oswald J., Yoshimatsu T., Franze K., Harris W.A. (2015). Müller glia provide essential tensile strength to the developing retina. J Cell Biol.

[16] Taylor L., Moran D., Arnér K., Warrant E., Ghosh F. (2013). Stretch to see: lateral tension strongly determines cell survival in long-term cultures of adult porcine retina. Invest Ophthalmol Vis Sci.

[17] Lindqvist N., Liu Q., Zajadacz J., Franze K., Reichenbach A. (2010). Retinal glial (Müller) cells: sensing and responding to tissue stretch. Invest Ophthalmol Vis Sci..

[18] Ruzafa N., Vecino E. (2015). Effect of Müller cells on the survival and neuritogenesis in retinal ganglion cells. Archivos de la Sociedad Española de Oftalmología (English Edition).

[19] Pereiro X., Ruzafa N., Azkargorta M., Elortza F., Acera A., Ambrósio A.F. (2024). Müller glial cells located in the peripheral retina are more susceptible to high pressure: implications for glaucoma. Cell Biosci.

[20] Guo L., Moss S.E., Alexander R.A., Ali R.R., Fitzke F.W., Cordeiro M.F. (2005). Retinal ganglion cell apoptosis in glaucoma is related to intraocular pressure and IOP-induced effects on extracellular matrix. Invest Ophthalmol Vis Sci.

[21] Li Q., Cheng Y., Zhang S., Sun X., Wu J. (2021). TRPV4-induced Müller cell gliosis and TNF-α elevation-mediated retinal ganglion cell apoptosis in glaucomatous rats via JAK2/STAT3/NF-κb pathway. J Neuroinflammation.

[22] Jo A.O., Lakk M., Rudzitis C.N., Križaj D. (2022). TRPV4 and TRPC1 channels mediate the response to tensile strain in mouse Müller cells. Cell Calcium.

[23] Prieto-López L., Pereiro X., Vecino E. (2024). The mechanics of the retina: müller glia role on retinal extracellular matrix and modelling. Front Med (Lausanne).

[24] Miller C.G., Budoff G., Prenner J.L., Schwarzbauer J.E. (2017). Minireview: fibronectin in retinal disease. Exp Biol Med (Maywood).

[25] Burke J.M., Kower H.S. (1980). Collagen synthesis by rabbit neural retina in vitro and in vivo. Exp Eye Res.

[26] Candiello J., Cole G.J., Halfter W. (2010). Age-dependent changes in the structure, composition and biophysical properties of a human basement membrane. Matrix Biol.

[27] Reinhard J., Wiemann S., Hildebrandt S., Faissner A. (2021). Extracellular matrix remodeling in the retina and optic nerve of a novel glaucoma mouse model. Biology (Basel).

[28] Fan J., Shen W., Lee S.-R., Mathai A.E., Zhang R., Xu G. (2020). Targeting the notch and TGF-β signaling pathways to prevent retinal fibrosis in vitro and in vivo. Theranostics.

[29] Ignotz R.A., Massagué J. (1986). Transforming growth factor-beta stimulates the expression of fibronectin and collagen and their incorporation into the extracellular matrix. J Biol Chem.

[30] Lu Y.-B., Franze K., Seifert G., Steinhäuser C., Kirchhoff F., Wolburg H. (2006). Viscoelastic properties of individual glial cells and neurons in the CNS. Proc Natl Acad Sci.

[31] Qu Y., He Y., Zhang Y., Ma T., Zhu J., Miao Y. (2018). Quantified elasticity mapping of retinal layers using synchronized acoustic radiation force optical coherence elastography. Biomed Opt Express.

[32] Qu Y., He Y., Saidi A., Xin Y., Zhou Y., Zhu J. (2018). In vivo elasticity mapping of posterior ocular layers using acoustic radiation force optical coherence elastography. Invest Ophthalmol Vis Sci.

[33] Last J.A., Pan T., Ding Y., Reilly C.M., Keller K., Acott T.S. (2011). Elastic modulus determination of normal and glaucomatous human trabecular meshwork. Invest Ophthalmol Vis Sci.

[34] Wang K., Johnstone M.A., Xin C., Song S., Padilla S., Vranka J.A. (2017). Estimating Human trabecular meshwork stiffness by numerical modeling and advanced OCT imaging. Invest Ophthalmol Vis Sci.

[35] Vahabikashi A., Gelman A., Dong B., Gong L., Cha E.D.K., Schimmel M. (2019). Increased stiffness and flow resistance of the inner wall of Schlemm's canal in glaucomatous human eyes. Proc Natl Acad Sci USA.

[36] Pereiro X., Ruzafa N., Acera A., Urcola A., Vecino E. (2020). Optimization of a method to isolate and culture adult porcine, rats and mice müller glia in order to study retinal diseases. Front Cell Neurosci.

[37] Moshayedi P., Da F., Costa L., Christ A., Lacour S.P., Fawcett J., Guck J. (2010). Mechanosensitivity of astrocytes on optimized polyacrylamide gels analyzed by quantitative morphometry. J Phys: Condens Matter.

[38] Bollmann L., Koser D.E., Shahapure R., Gautier H.O., Holzapfel G.A., Scarcelli G. (2015). Microglia mechanics: immune activation alters traction forces and durotaxis. Front Cell Neurosci.

[39] Hadjipanayi E., Mudera V., Brown R.A. (2009). Close dependence of fibroblast proliferation on collagen scaffold matrix stiffness. J Tissue Eng Regen Med.

[40] Ghosh K., Pan Z., Guan E., Ge S., Liu Y., Nakamura T. (2007). Cell adaptation to a physiologically relevant ECM mimic with different viscoelastic properties. Biomaterials.

[41] Rheinlaender J., Dimitracopoulos A., Wallmeyer B., Kronenberg N.M., Chalut K.J., Gather M.C. (2020). Cortical cell stiffness is independent of substrate mechanics. Nat Mater.

[42] Bu S.-C., Kuijer R., van der Worp R.J., van Putten S.M., Wouters O., Li X.-R. (2015). Substrate elastic modulus regulates the morphology, focal adhesions, and α-smooth muscle actin expression of retinal Müller cells. Invest Ophthalmol Vis Sci.

[43] Kanda A., Noda K., Hirose I., Ishida S. (2019). TGF-β-SNAIL axis induces Müller glial-mesenchymal transition in the pathogenesis of idiopathic epiretinal membrane. Sci Rep.

[44] Krishna Chandran A.M., Coltrini D., Belleri M., Rezzola S., Gambicorti E., Romano D. (2021). Vitreous from idiopathic epiretinal membrane patients induces glial-to-mesenchymal transition in Müller cells. Biochimica et Biophysica Acta (BBA) - Molecular Basis of Disease.

[45] Ryskamp D.A., Frye A.M., Phuong T.T.T., Yarishkin O., Jo A.O., Xu Y. (2016). TRPV4 regulates calcium homeostasis, cytoskeletal remodeling, conventional outflow and intraocular pressure in the mammalian eye. Sci Rep.

[46] Sianati S., Schroeter L., Richardson J., Tay A., Lamandé S.R., Poole K. (2021). Modulating the mechanical activation of TRPV4 at the cell-substrate interface. Front Bioeng Biotechnol.

[47] Moeendarbary E., Weber I.P., Sheridan G.K., Koser D.E., Soleman S., Haenzi B. (2017). The soft mechanical signature of glial scars in the central nervous system. Nat Commun.

[48] Tang V.W. (2020). Collagen, stiffness, and adhesion: the evolutionary basis of vertebrate mechanobiology. Mol Biol Cell.

[49] Swift J., Ivanovska I.L., Buxboim A., Harada T., Dingal P.C.D.P., Pinter J. (2013). Nuclear lamin-A scales with tissue stiffness and enhances matrix-directed differentiation. Science.

[50] Vecino E., Rodriguez F.D., Ruzafa N., Pereiro X., Sharma S.C. (2016). Glia–neuron interactions in the mammalian retina. Prog Retin Eye Res.

[51] Jennings J.C., Mohan S., Linkhart T.A., Widstrom R., Baylink D.J. (1988). Comparison of the biological actions of TGF beta-1 and TGF beta-2: differential activity in endothelial cells. J Cell Physiol.

[52] Hoerster R., Muether P.S., Vierkotten S., Hermann M.M., Kirchhof B., Fauser S. (2014). Upregulation of TGF-ß1 in experimental proliferative vitreoretinopathy is accompanied by epithelial to mesenchymal transition. Graefes Arch Clin Exp Ophthalmol.

[53] Fuchshofer R., Tamm E.R. (2012). The role of TGF-β in the pathogenesis of primary open-angle glaucoma. Cell Tissue Res.

[54] Inman G.J., Nicolás F.J., Callahan J.F., Harling J.D., Gaster L.M., Reith A.D. (2002). SB-431542 is a potent and specific inhibitor of transforming growth factor-beta superfamily type I activin receptor-like kinase (ALK) receptors ALK4, ALK5, and ALK7. Mol Pharmacol.

[55] Bureta C., Setoguchi T., Saitoh Y., Tominaga H., Maeda S., Nagano S. (2019). TGF-β promotes the proliferation of microglia In vitro. Brain Sci.

[56] Rozovsky I., Finch C.E., Morgan T.E. (1998). Age-related activation of microglia and astrocytes: in vitro studies show persistent phenotypes of aging, increased proliferation, and resistance to down-regulation. Neurobiol Aging.

[57] Suzumura A., Sawada M., Yamamoto H., Marunouchi T. (1993). Transforming growth factor-beta suppresses activation and proliferation of microglia in vitro. J Immunol.

[58] Zhang J., Zhang L., Yi S., Jiang X., Qiao Y., Zhang Y. (2020). Mouse astrocytes promote microglial ramification by releasing TGF-β and forming glial fibers. Front Cell Neurosci.

[59] Enzo M.V., Cattelan P., Rastrelli M., Tosi A., Rossi C.R., Hladnik U. (2019). Growth rate and myofibroblast differentiation of desmoid fibroblast-like cells are modulated by TGF-β signaling. Histochem Cell Biol.

[60] Merrilees M.J., Sodek J. (1992). Synthesis of TGF-beta 1 by vascular endothelial cells is correlated with cell spreading. J Vasc Res.

[61] Wilson S.E. (2021). TGF beta −1, −2 and −3 in the modulation of fibrosis in the cornea and other organs. Exp Eye Res.

[62] Grande J.P., Melder D.C., Zinsmeister A.R. (1997). Modulation of collagen gene expression by cytokines: stimulatory effect of transforming growth factor-β1, with divergent effects of epidermal growth factor and tumor necrosis factor-α on collagen type I and collagen type IV. J Lab Clin Med.

[63] Sappington R.M., Chan M., Calkins D.J. (2006). Interleukin-6 protects retinal ganglion cells from pressure-induced death. Invest Ophthalmol Vis Sci.

[64] Inman G.J., Nicolás F.J., Callahan J.F., Harling J.D., Gaster L.M., Reith A.D. (2002). SB-431542 is a potent and specific inhibitor of transforming growth factor-β superfamily type I activin receptor-like kinase (ALK) receptors ALK4, ALK5, and ALK7. Mol Pharmacol.

[65] Schneider C.A., Rasband W.S., Eliceiri K.W. (2012). NIH image to ImageJ: 25 years of image analysis. Nat Methods.

