# Supplementary figures and images for: Substrate stiffness and pressure alter retinal Müller glia response and extracellular matrix production
Source: Biomater Biosyst. 2025 Jul 7;19:100114. doi: 10.1016/j.bbiosy.2025.100114 (PMC12275231; doi:10.1016/j.bbiosy.2025.100114)

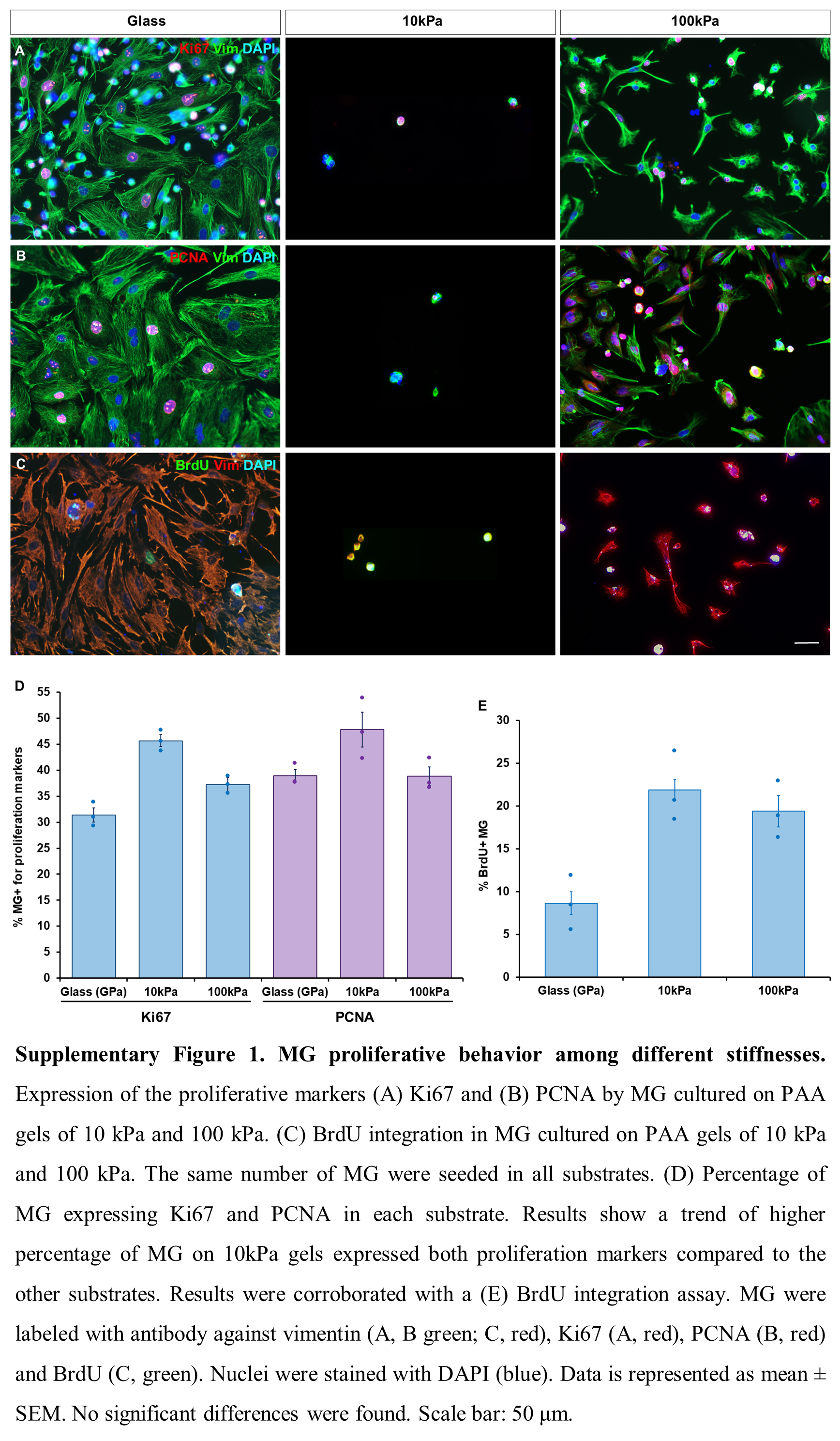

Supplement: Supplementary file 1 [file mmc1.zip › Fig. S1.tif]

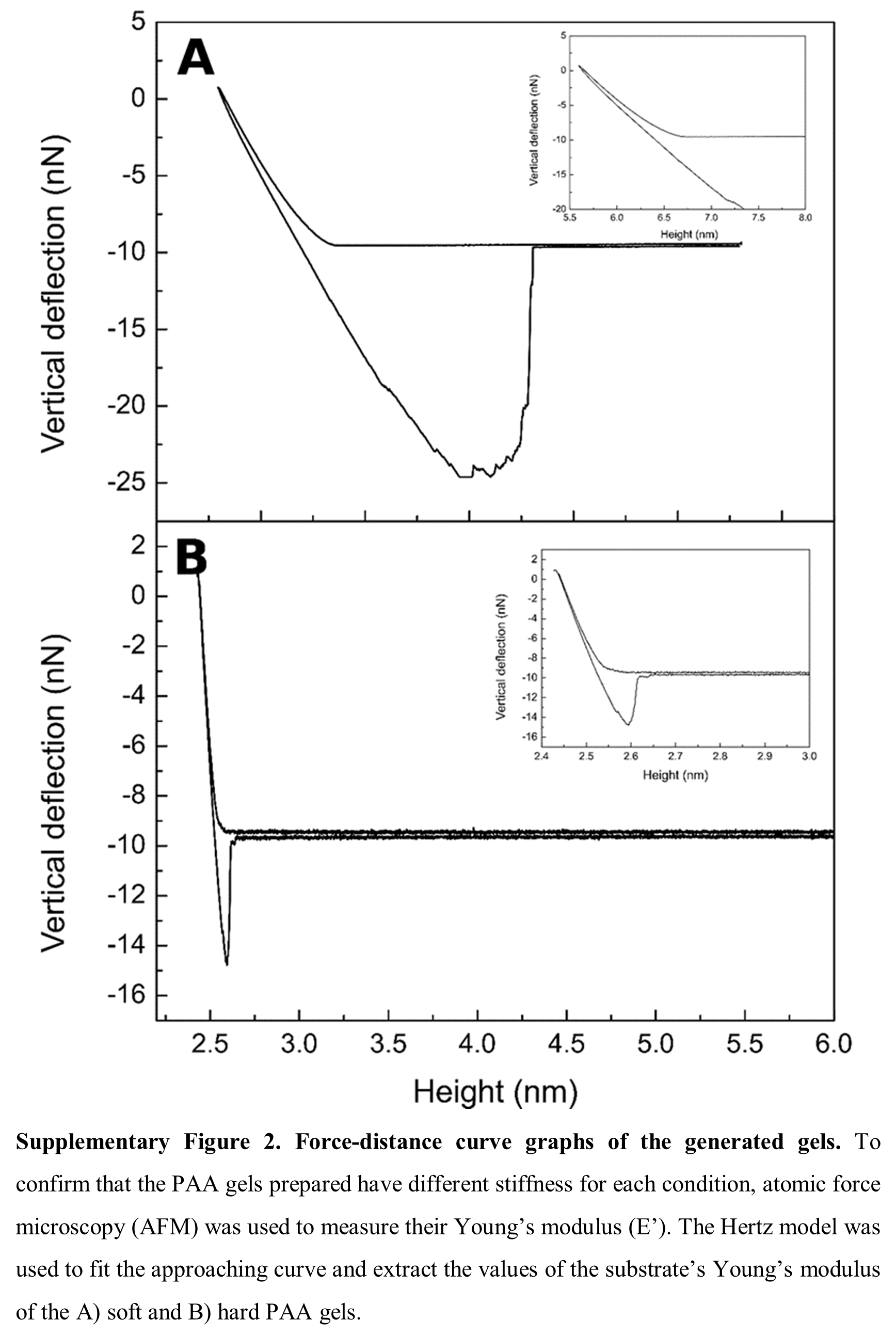

Supplement: Supplementary file 2 [file mmc2.zip › Fig. S2.tif]
